# Nelarabine-induced rhabdomyolysis in a patient with T-cell acute lymphoblastic leukemia: a case report

**DOI:** 10.1186/s40780-022-00247-w

**Published:** 2022-06-11

**Authors:** Akari Utsumi, Yuri Goto, Takaaki Suzuki, Chiaki Imai, Shinichiro Matsui, Emiko Sakaida, Itsuko Ishii

**Affiliations:** 1 Division of Pharmacy, Chiba University Hospital, 1-8-1 Inohana Chuo-ku, Chiba, 260-8677 Japan; 2 Division of Hematology, Department of Clinical Cell Biology, Chiba University Graduate School of Medicine, Chiba, Japan

**Keywords:** Rhabdomyolysis, Nelarabine, Creatine kinase

## Abstract

**Background:**

Nelarabine is an antineoplastic purine analog used for the treatment of refractory or relapsed T-cell acute lymphoblastic leukemia (T-ALL). The most prominent side effect of nelarabine are neurotoxicity and hematologic disorder, which are considered dose-limiting factors. Although clinical studies have reported myopathy due to nelarabine, actual detailed outcomes were not well-known initial approval. The incidence of nelarabine induced rhabdomyolysis has been reported at 2% in study in children. Cases of rhabdomyolysis have been reported in adults from medical facilities in the United Sates with renal dysfunction or severe muscle symptoms after administration of multiple courses of nelarabine. In this report, we discuss a case of rhabdomyolysis diagnosed after a single course of nelarabine. In this case, creatine kinase (CK) level was elevated in grade 4, without renal dysfunction and severe muscle symptoms.

**Case presentation:**

A 46-year-old man from Japan was diagnosed with T-ALL and received a hematopoietic stem cell transplantation in first remission. However, the disease relapsed 6 months after transplantation. Nelarabine was selected as the next-line chemotherapeutic agent. The patient received 1500 mg/m^2^ of nelarabine on day 1 followed by a dose on days 3 and 5. CK levels, which were baseline before treatment, increased to grade 4 (18,620 IU/L) on the 8th day of treatment. He was diagnosed as rhabdomyolysis due to nelarabine with little possibility of other factors. He complained only of mild pain in his upper extremities and no other symptoms were noticed. The patient was managed with hydration. The pain lasted approximately 7 days, but there were no sequelae secondary to the rhabdomyolysis. Because of the elevation of CK in grade 4, we avoided re-administration.

**Conclusion:**

In the patient administrated nelarabine, CK level was elevated in grade 4, without other symptoms of rhabdomyolysis. The results suggest that CK may be elevated at the onset of rhabdomyolysis caused by nelarabine, even in the absence of other symptoms. Therefore, it was suggested that monitoring CK during nelarabine administration is important for detecting rhabdomyolysis before it becomes severe. We consider that CK should be monitored even in absence of symptoms.

## Introduction

Nelarabine is an anticancer purine analog used in the treatment of refractory or relapsed T-cell acute lymphoblastic leukemia (T-ALL). Neuropathy is well known as an important side effect, but myotoxicity is less well known [1, 2]. The PGAA2002 phase II trial, conducted in the United States (U.S.), reported that muscle pain occurred in 13% of patients, with 1 patient (1% of all participants) developing grade 3 symptoms [3]. There was no description of rhabdomyolysis. After marketing, side effect such as elevated creatine kinase (CK) and rhabdomyolysis [4–7] were reported. The COGALL0434 trial targeting children reported that rhabdomyolysis occurred in 2% of patients who received nelarabine in consolidation therapy [4, 5]. Some patients in this study received nelarabine before consolidation therapy, and it is not known how many doses of nelarabine they received before developing rhabdomyolysis. Two case reports of nelarabine induced rhabdomyolysis from medical facilities in the U.S. reported onset after the second [6] and third courses [7] of treatment. They were discovered through muscle symptoms. Since rhabdomyolysis is a fatal side effect, caution should be exercised when administering nelarabine, but its period likely to occur and information on approaches to avoid serious condition are not yet well known.

## Case

A 46-year-old Japanese man presented with exertional dyspnea and malaise and was diagnosed with T-ALL in February 2019. The bone marrow was occupied by 99% lymphoblast-like cells. Immunophenotype (cyCD3+, TdT+, CD19-) supported the diagnosis. And the results of G-band staining showed a complex karyotype (47, XY, del (6)(q?), − 7, − 13, − 14, +mar1, +mar2, +mar3, +mar4[20/20]), which predicted a poor prognosis. A complete blood count showed hemoglobin of 4.7 g/dL, platelet count of 90,000/μL, and white blood cell count of 17,000/μL with 87% blast cells.

Remission induction therapy was started with the Japan Adult Leukemia Study Group (JALSG)-ALL 202-O protocol [8]: cyclophosphamide 1.2 g/m^2^ on day 1, daunorubicin 60 mg/m^2^ on days 1, 2, and 3, vincristine 1.3 mg/m^2^ on days 1, 8, 15, and 22, L-asparaginase 3000 U/m^2^ on days 9, 11, 13, 16, 18, and 20, and prednisolone 60 mg/m^2^ from days 1 to 21. However, the treatment could not continue after day 10 because the patient developed septic shock. After the patient recovered, JALSG-ALL 202-O Consolidation Therapy Course 1 was started with the dose adjusted according to the patient’s liver function: cytarabine 2 g/m^2^ every 12 h for 6 doses, etoposide 100 mg/m^2^ from days 1 to 3, and dexamethasone 40 mg from days 1 to 3. This chemotherapy was administered for 3 courses. During the treatment course, the patient underwent left upper lobectomy to remove an abscess. Due to the chemotherapy, the patient achieved complete remission.

Given his remission status, the patient received hematopoietic stem cell transplantation after conditioning with fludarabine (30 mg/m^2^ for 6 days), melphalan (70 mg/m^2^ for 2 days), and 2 Gy of total body irradiation in July 2019. As complications, bacteremia caused by *Pseudomonas putida*, pneumonia of unknown etiology, and grade 2 intestinal graft-versus-host disease developed. When variable number of tandem repeat analysis confirmed 100% hematopoietic reconstitution, he was discharged 91 days after transplantation. Unfortunately, the patient relapsed 6 months after the hematopoietic stem cell transplantation. Subsequent chemotherapy comprised high-dose ara-C (cytarabine 2 g/m^2^ for 5 days) and inotuzumab ozogamicin. However, remission was not achieved with these treatments and nelarabine was selected as the next-line chemotherapeutic agent.

**Fig. 1:**
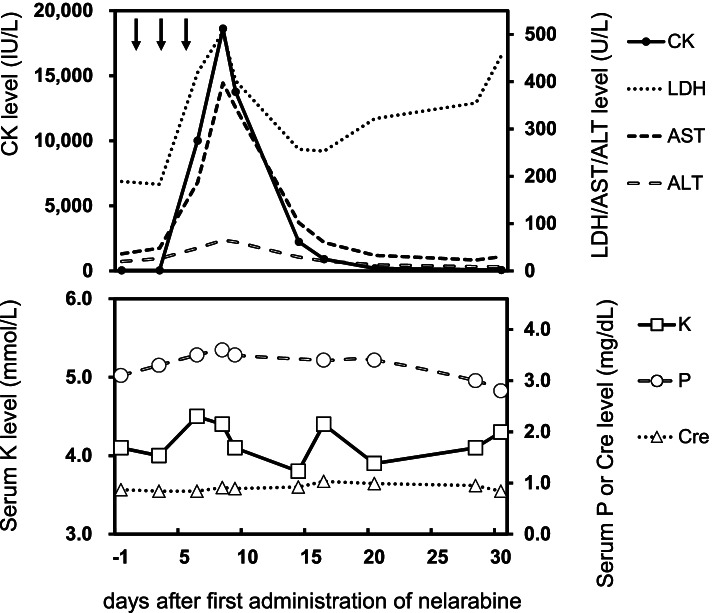
CK levels after the first cycle of nelarabine administration in our patient. Nelarabine was administered on days 1, 3, and 5. Arrows show the days of nelarabine administration. The CK level was 48 IU/L before nelarabine administration, reached 18,620 IU/L on day 8, and returned to baseline on day 38. ALT, alanine transaminase; AST, aspartate transaminase; CK, creatine kinase; LDH, lactate dehydrogenase. K, potassium; P, phosphorus; Cre, creatinine

In April 2020, the patient received 1500 mg/m^2^ of nelarabine on day 1 followed by a dose on days 3 and 5. However, on day 6, his CK level, previously within the normal range (40 IU/L), increased to 10,008 IU/L and the patient reported tenderness in the left upper arm. The CK level reached 18,620 IU/L on day 8 (Fig. 1). Other laboratory findings showed that the serum lactate dehydrogenase, aspartate transaminase, and alanine transaminase levels were increased to 505 U/L, 397 U/L, and 65 U/L, respectively. Their respective levels had previously been normal at 192 U/L, 30 U/L, and 17 U/L. No increase in creatinine (Cre) was observed and it remained at about 0.9 mg/dL. Potassium and phosphorus level remained roughly flat and within reference values. Since there was no change in urinary characteristics during the interview, a urinalysis was not performed. Because the patient did not report cardiac pain, we did not measure creatine phosphokinase-MB isozyme levels. Rhabdomyolysis was diagnosed, the patient was managed with hydration. The pain lasted approximately 7 days, but there were no sequelae secondary to the rhabdomyolysis.

Although the nelarabine therapy was effective, it was not possible to continue the treatment because of the patient’s grade 4 CK elevation. The patient then underwent chemotherapy with clofarabine and hematopoietic stem cell transplantation. Eventually, he died of hepatic veno-occlusive disease after the transplantation.

Our patient was diagnosed with rhabdomyolysis after first-cycle nelarabine administration. He did not have well-known typical causes of rhabdomyolysis, such as trauma, extreme physical activity, and prolonged muscle compression. Infection is also one of the common causes of rhabdomyolysis, and the patient had a history of lung abscess and septic shock. However, this was not considered the main cause because the infection was not active and his body temperature was 36.6 °C and C-reactive protein level was 0.61 mg/dL on the day of the CK peak.

Concomitant medications on the day of nelarabine administration included trimethoprim-sulfamethoxazole, voriconazole, esomeprazole magnesium hydrate, and acyclovir. They were administered at least 1 month prior to the start of nelarabine and were continued after nelarabine started, without CK elevation. Granisetron was also administered on the day of nelarabine administration. It had been used previously without CK elevation. These drugs were continued or redosed after the present event and there were no signs of rhabdomyolysis. In addition, these agents are not generally known as high-risk drug of rhabdomyolysis. They are thus improbable causes of the rhabdomyolysis.

## Discussion and conclusion

We attribute the patient’s rhabdomyolysis to nelarabine administration because of the chronological and temporal sequence of events and lack of any other potential factors. According to the Naranjo adverse drug reaction probability scale [9], nelarabine would score ~ 6, which would make it the “probable” cause of the adverse reaction.

In the previously reported two cases, rhabdomyolysis was found due to complaints of myalgia and muscle weakness. Haider M. et al. reported a case with muscle pain so bad incapacitating on day 7 of second-cycle nelarabine administration [6]. Cre and electrolytes were unchanged at this time. Pandey RK. et al. reported a case required emergent dialysis [7]. CK and Cre level were within reference values on day 3, but he had progressive myalgia symptoms starting on day 5, and his CK and Cre level were elevated in grade 4 on day 8, after 3rd course of administration. Although the CK elevation in our patient was grade 4, as in the previously reported case, our patient’s muscle pain was mild and Cre was stable, unlike in the previously reported cases. This case suggests that CK may be elevated at the onset of rhabdomyolysis by nelarabine administration without leading to muscle symptoms or elevated Cre level. Since we thought that rhabdomyolysis had occurred when the CK level increased, it is probable that the patient did not develop muscle symptoms or Cre level elevation due to hydration and discontinuation of the suspected drug. Therefore, it was suggested that monitoring CK during nelarabine administration could detect rhabdomyolysis before it becomes severe.

The CK level of our patient peaked on day 8 and that of the previously reported patients peaked around day 8. Rhabdomyolysis due to nelarabine is likely to develop a few days after administration of nelarabine for 5 days. The patient received nelarabine after transplantation, but the previously reported patients had not undergone transplantation [6, 7]. Nelarabine has a risk of rhabdomyolysis with or without a history of transplantation.

Nelarabine is one of the treatment options for relapsed and refractory T-ALL in remission therapy and consolidation therapy for hematopoietic stem cell transplantation. Rhabdomyolysis is defined as “a state of muscle injury that can lead to several forms of systemic insult, such as acute kidney injury, electrolyte imbalance, and disseminated intravascular coagulation” [10]. The intensive care unit mortality of patients with rhabdomyolysis and acute renal failure is reported to be 59%. If a patient develops rhabdomyolysis and severe kidney injury, their treatment options will be narrowed and they will be unable to undergo transplantation. Therefore, severe rhabdomyolysis may have a significant impact on treatment prognosis. Renal dysfunction due to rhabdomyolysis is thought to be caused by multiple factors such as myoglobulin leaked from the myocytes as they are destroyed. Serum CK levels are said to begin to rise within 2–12 hours after myocyte damage and are thought to be observed prior to renal dysfunction [10]. This is consistent with the phenomenon observed in the present case. Causative drugs should be detected early and promptly discontinued to prevent the development of severe symptoms. We consider that the monitoring of subjective symptoms alone is insufficient and that CK monitoring is important to detect rhabdomyolysis early.

In adults, drugs are a well-known and common cause of rhabdomyolysis, but anti-cancer drug-induced rhabdomyolysis is not common. Although this condition has been reported with oxaliplatin [11] and cytarabine [12], the underlying pathogenic mechanisms are unclear. The pathogenic mechanism of nelarabine-induced rhabdomyolysis is also not known. Nelarabine is a prodrug that is converted to 9-β-D-arabinofuranosylguanosine (ara-G) by adenine deaminase in the blood, which is then taken up into cells through a transporter. After being metabolized to ara-G triphosphate, it induces apoptosis by inhibiting deoxyribonucleic acid (DNA) synthesis and repair [13]. Because skeletal muscle cells do not divide, nelarabine is not considered to damage the tissue normally. However, when skeletal muscle is damaged by exercise, pressure necrosis, or other causes, satellite cells are activated and differentiate into myoblasts to proliferate [14]. Administration of nelarabine during myoblast cell division is believed to cause muscle repair failure and damage to muscle fibers. The patient did not develop rhabdomyolysis after treatment with fludarabine or clofarabine, which have similar structures and mechanisms of action as nelarabine. Therefore, it is unlikely that this is a risk associated with purine analogs, and care should be taken in the timing of administration, such as avoiding administration after exercise.

In the patient administrated nelarabine, CK level was elevated in grade 4, without symptoms of rhabdomyolysis. The results suggest that CK may be elevated at the onset of rhabdomyolysis caused by nelarabine, even in the absence of other symptoms. Therefore, it was suggested that monitoring CK during nelarabine administration is important for detecting rhabdomyolysis before it becomes severe. We consider that CK should be monitored even in the absence of symptoms. In addition, monitoring should be continued at least until around day 8, even if it’s the first cycle of nelarabine.

## Data Availability

Not applicable.

## Abbreviations

T-ALL: T-cell acute lymphoblasticleukemia
CK: Creatine kinase
JALSG: Japan Adult Leukemia Study Group
Cre: Creatinine
ara-G: 9-β-D-arabinofuranosylguanosine
DNA: Deoxyribonucleic acid
